# Supplementary material for: Can Model Experiments Give Insight into the Response of the Soil Environment to Flooding? A Comparison of Microcosm and Natural Event
Source: Biology (Basel). 2022 Mar 1;11(3):386. doi: 10.3390/biology11030386 (PMC8945539; doi:10.3390/biology11030386)
Supplement: Supplementary file 1 [file biology-11-00386-s001.zip › biology-1583651-supplementary.pdf]

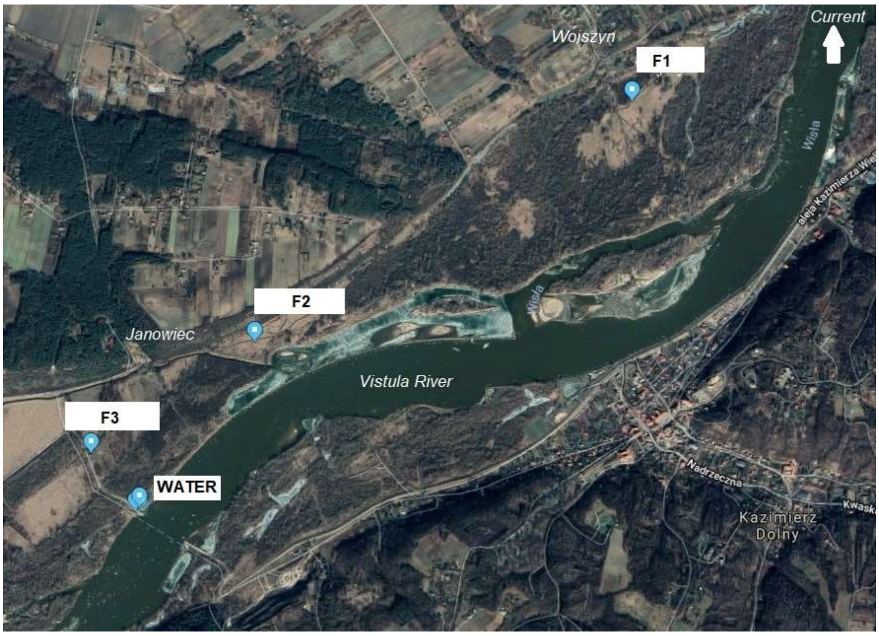

Figure S1. Location of sampling area. Explanation of the samples in Table 1. For more details about this location, see Figure 1 in Furtak et al. (2020) [21].

Table S1. Selected soil characteristics parameters for fluvisols used in the experiment (depth 0–20 cm); according to Furtak et al. (2020) [21].

Fluvisol	Soil texture, mm [%]			Textural classes USDA[1]	RDC [g 100 g^{-1} soil]	Humus [%]
	2.0-0.05	0.05-0.002	<0.002			
**F1**	58	38	4	SL	0.16 ^{a}	4.74 ± 0.06 ^{a}
**F2**	67	30	3	SL	0.32 ^{b}	2.94 ± 0.03 ^{b}
**F3**	92	8	0	S	*n.d.*[2]	0.95 ± 0.04 ^{c}

[1] According to the USDA classification: SL – sandy loam, S – sand. RDC—readily dispersible clay.
[2] The content of RDC was not determined in F3 because the content (%) of colloidal clay (soil texture < 0.002 mm) in the sample was 0.00; means ± standard deviation (SD); different letters (a–c) in the columns indicate values with significant difference at $P < 0.05$ (n=3) by Tukey's honest significant difference (HSD) test.
Explanation of the samples in Table 1.

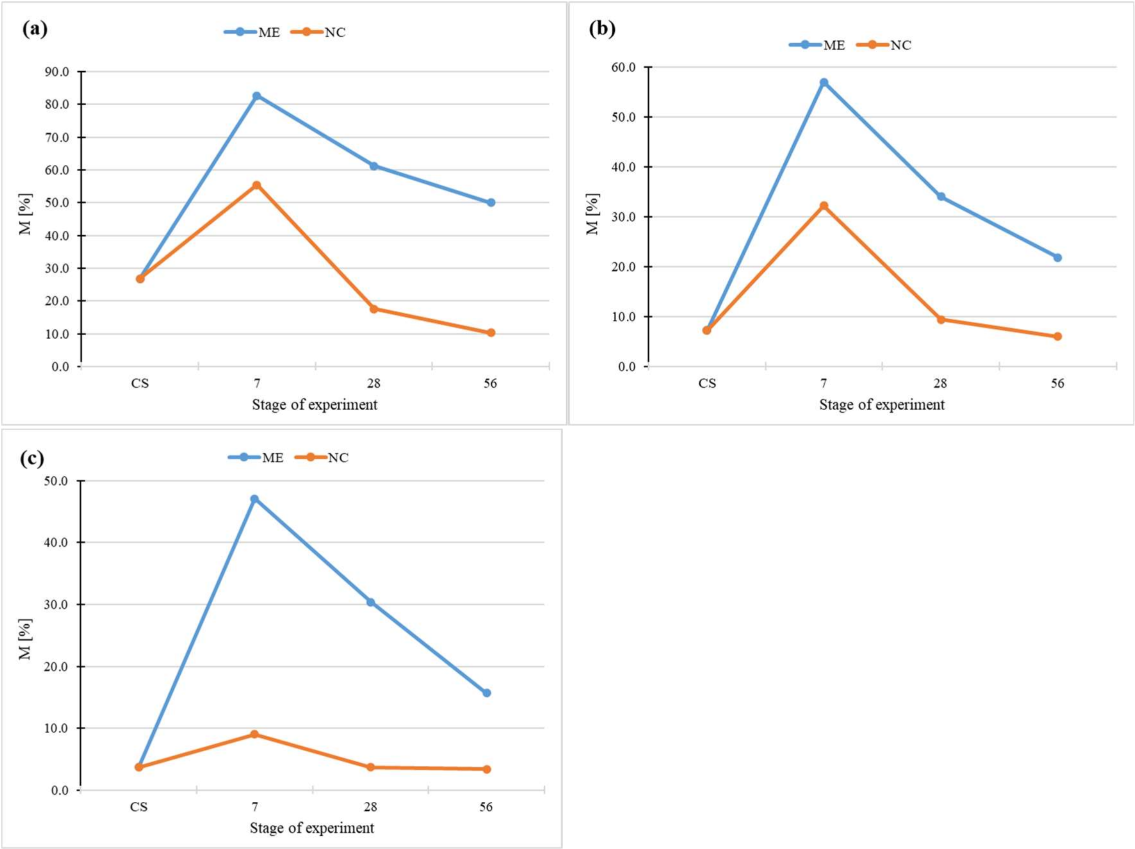

Figure S2. Soil moisture (%) contents in different experimental conditions. (**a**) F1; (**b**) F2; (**c**) F3. Measured using gravimetric (drying) method.. Explanation of the samples in Table 1.
